# A mixture of *Pueraria lobata* and *Platycodon grandiflorum* extracts ameliorates RANKL-induced osteoclast differentiation and ovariectomy-induced bone loss by regulating Src- PI3K-AKT and JNK/p38 signaling pathways

**DOI:** 10.1016/j.heliyon.2024.e24842

**Published:** 2024-01-17

**Authors:** Jisun Song, Suhyun Han, Sooyeon Choi, Jungkyu Lee, Yoonseon Jeong, Hyun Myung Lee, JongDai Son, Dam Yeon Jeong, Seung-Shin Yu, Wonwoo Lee

**Affiliations:** R&D Center for Innovative Medicines, Helixmith Co., Ltd., Seoul 07794, South Korea

**Keywords:** *Pueraria lobata*, *Platycodon grandiflorum*, Herbal extracts, Osteoclast, Osteoporosis

## Abstract

Osteoporosis is caused by increased bone resorption due to the excessive activity of osteoclasts. *Pueraria lobata* has demonstrated the ability to improve bone density in ovariectomized mice, and *Platycodon grandiflorum* can suppress osteolysis biomarkers such as collagen content in cartilage and alkaline phosphatase activity. In this study, we examined whether HX112, a mixture of *Pueraria lobata* and *Platycodon grandiflorum* extracts, could inhibit the receptor activator of nuclear factor-κB (NF-κB) ligand (RANKL)-induced osteoclast differentiation to alleviate osteoporosis. To induce the differentiation of osteoclasts, RAW 264.7 cell were cultured with RANKL and HX112. Osteoclasts differentiation was evaluated by TRAP activity and TRAP staining. Bone resorption as osteoclasts major function was assessed by pit formation assay. As a result, HX112 suppressed osteoclast differentiation and bone resorptive function. Additionally, HX112 reduced the expression of osteoclastogenic genes including NFATc1 and c-Fos, and these effects of HX112 were mediated by inhibiting Src-phosphoinositide 3-kinase (PI3K)- Protein kinase B (Akt) and c-Jun N-terminal kinase (JNK)/p38 signaling pathways. Furthermore, ICR mice were ovariectomized to induce osteoporosis and bone mineral density of femur was measured using micro-CT. Consequently, oral administration of HX112 to ovariectomized mice significantly improved bone microstructure and bone mineral density. Collectively, these findings indicate that the mixed extract of *Pueraria lobata* and *Platycodon grandiflorum* may be useful as therapeutics for osteoporosis.

## 1 Introduction

Osteoporosis is one of the skeletal diseases that reduce bone strength and increase the chance of fracture [1,2]. Patients with osteoporosis have lower bone density than the average of young women by 2.5 standard deviations or more [3,4]. Recently, the global prevalence of osteoporosis has been reported to be 18.3%, and it is estimated that the prevalence will increase as the population ages (30% for 50–60 years old and over 50% for 80 years old) [5,6]. Drugs currently used to treat osteoporosis include denosumab and bisphosphonates such as alendronate, ibandronate, and zoledronate [7,8]. However, the long-term use of these medications can reduce patient quality of life due to various side effects such as difficulty swallowing, esophageal inflammation, gastric ulcers, and joint or muscle pain [7]. Therefore, to address these medical unmet needs, novel therapeutic agents for osteoporosis without safety concerns need to be developed.

Bone remodeling refers to the process by which old bone is decomposed and new bone is synthesized; this mechanism is regulated by osteoclasts and osteoblasts. When osteoclasts are more active than osteoblasts, the balance of bone remodeling is impaired, resulting in the development of osteoporosis [9,10]. Osteoclasts formed by mononuclear cells fusion have a bone resorption function [11]. Osteoclasts acidify the microenvironment of the bone resorption site through proton pumps and release various proteases to degrade and resorb the bone [10].

Several factors influence osteoclast differentiation. In particular, the receptor activator of nuclear factor-κB (NF-κB) ligand (RANKL) is considered an essential cytokine in regulating osteoclast formation [11,12]. Numerous studies have shown that when RANKL binds to the receptor activator of NF-κB (RANK), signaling pathways including phosphoinositide 3-kinase (PI3K), mitogen-activated protein kinase (MAPK), and NF-κB, are activated [11,13]. The PI3K signaling pathway is a widely recognized pathway that plays a role in osteoclastogenesis and bone resorption. For example, treating cells with the PI3K inhibitor LY294002 or wortmannin restrained osteoclast generation [[14], [15], [16]]. Additionally, mice lacking the Src homology 2 domain–containing inositol polyphosphate 5′-phosphatase (SHIP) exhibited negatively regulated PI3K signaling, increased number of osteoclasts, and severe osteoporosis. Another signaling pathway thought to be involved in osteoclast differentiation is the MAPK signaling pathway [13,17]. A previous study showed that RANKL-mediated osteoclastogenesis were inhibited in bone marrow (BM) cells from ERK1-deficient mice [18]. Moreover, there have also been reports that SP600125, JNK inhibitor, treatment significantly suppressed osteoclast differentiation in BM cells [19,20]. Furthermore, SB203580, a p38-specific inhibitor, decreased the differentiation of osteoclasts by regulation of nuclear factor of activated T cells 1 (NFATc1) and c-Fos expression [15,[21], [22], [23]]. Collectively, these findings showed that ERK, JNK, and p38 signaling activities of the MAPK family are closely related to the differentiation of osteoclast.

HX112 is a mixture of herbal extract from *Pueraria lobata* and *Platycodon grandiflorum*. *P*. *lobata* is an edible herb in the Leguminosae family that has long been used as a remedy for cardiovascular diseases and diabetes, and also osteoporosis and hot flushes in postmenopausal women [[24], [25], [26], [27], [28], [29]]. More than 70 chemical components have been identified in *P. lobata*, including isoflavones, triterpenes, coumarins, and puerarols. Among them, the isoflavone content is the highest, and puerarin, daidzein, and daidzin are the most abundant isoflavone components [24]. The isoflavonoids present in *P*. *lobata* have anti-inflammatory and antioxidant properties [30,31]. In addition, ovariectomized mice fed *Puerariae radix* or isoflavones isolated from *P. lobata* showed improved femur bone mineral density (BMD) [32,33]. Recently, it was revealed that *P*. *lobata* ethanol extract inhibits the differentiation and activity of osteoclasts through the MAPK signaling pathway [34]. *P*. *grandiflorum* belongs to the Campanulaceae family and has anti-inflammatory, antioxidant, antitumor, and antitussive properties [[35], [36], [37], [38], [39], [40]]. Saponins extracted from *P. grandiflorum* induced the differentiation of osteoblasts through the p38 and ERK pathways [41]. Based on these studies, we speculated that combination of *P. lobata* and *P. grandiflorum* may regulate the balance of osteoclasts and osteoblasts, potentially resulting in higher osteoporosis improvement efficacy than single extracts. Therefore, we hypothesized that HX112, a mixture of *P. lobata* and *P. grandiflorum*, has the potential to be a candidate for the treatment of osteoporosis by inhibiting the differentiation and function of osteoclasts, which play major role in causing osteoporosis, and improving bone density.

In this study, we found that HX112 restrains osteoclast differentiation and bone resorption mediated through RANKL. Additionally, HX112 decreased the expression of osteoclastogenic genes by blocking Src-PI3K-Akt and JNK/p38 signaling pathways. Furthermore, administering HX112 to ovariectomized mice improved bone microstructure and BMD. These results suggest that HX112 may be effective in preventing osteoporosis by inhibiting osteoclast differentiation and bone resorption.

## 2 Materials and methods

### 2.1 HX112 extract preparation and high-performance liquid chromatography (HPLC) analysis

The components of HX112, *P*. *lobata* and *P*. *grandiflorum*, were obtained from Omnihub (Korea) and genetically classified. HX112 was made by mixing *P*. *lobata* and *P*. *grandiflorum* at a 1:1 ratio. The mixture was extracted with 25 % aqueous ethanol for 72 h at room temperature. After filtering the extract through a 5 μm cartridge, it was concentrated with a rotary evaporator and then subjected to spray drying with dextrin. The extraction yield was approximately 30 %, and voucher specimens were kept at the R&D center of Helixmith Co., Ltd. (Korea). Voucher Specimen NO: D1701245031K for *Pueraria lobata* (Wild.) Ohwi; D180103120K for *Platycodon grandiflorum* (Jacq.) A.DC.

The chemical characteristics of HX112 were identified using two methods. The components derived from *P*. *lobata* were analyzed using HPLC-photo diode array (Waters Alliance e2695 system and 2998 detector; USA), and the components derived from *P. grandiflorum* were analyzed using HPLC-evaporative light scattering detector (ELSD; Waters Alliance e2695 system and 2424 detector; USA). Supplementary Material present the HPLC analysis condition details (Supplementary table 1 and 2).

### 2.2 Cell culture

RAW264.7 was obtained from ATCC (TIB-71; USA). The cells were cultured in Dulbecco's modified Eagle's medium containing 1 % l-glutamine, streptomycin 100 μg/mL, penicillin 100 units/mL, and 10 % heat-inactivated fetal bovine serum (all from Gibco; USA).

### 2.3 Cell viability

Cell viability assay was performed using the Enhanced Cell Viability Assay Kit (Abfrontier, Korea). Briefly, HX112-treated cells were cultured with WST-1 for 30 min, after which the absorbance was evaluated using a VARIOSKAN LUX Multimode microplate reader (Thermo Fisher, USA) at wavelengths of 450–630 nm.

### 2.4 Tartrate-Resistant acid phosphatase (TRAP) staining and activity

TRAP is an iron-containing enzyme expressed in osteoclasts and macrophages, and has been used as a histochemical marker for osteoclasts for a long time because it plays an important role in bone mineralization [42]. Therefore, we verified osteoclast differentiation through TRAP staining and activity assay. The TRAP assay is the most widely used method to detect differentiation of osteoclast populations in vitro [43]. Staining to identify TRAP positive cells was conducted using an acid phosphatase leukocyte kit (Sigma, USA). In brief, cells were cultured for 24 h in a minimum essential phenol red-free medium supplemented with 10 % charcoal-stripped fetal bovine serum and 1 % penicillin/streptomycin (all from Gibco). Thereafter, cells were exposed with varying concentrations of HX112 (50, 100, 200, or 400 μg/mL) and 50 ng/mL RANKL (R&D Systems, USA) for 6 d. Fixed cells were stained with TRAP solution. Osteoclasts were identified by counting multinucleated cells with three or more nuclei. To evaluate TRAP activity, TRAP solution buffer was produced according to the previously described method [44]. After RAW264.7 cells incubated with HX112 and RANKL differentiated into osteoclasts, treated with TRAP solution buffer at 37°C for 30 min. TRAP activity was determined using VARIOSKAN LUX Multimode microplate reader (Thermo Fisher, USA).

### 2.5 Pit formation assay

Pit formation was analyzed using a bone resorption assay kit (Cosmo Bio, Japan). RAW264.7 cells were cultured on calcium phosphate-coated plates and then stimulated with HX112 and RANKL for 12 d. After the cells were removed by 5 % sodium hypochlorite, the pits were observed under a light microscope and the area of each pit was evaluated by Image J software.

### 2.6 Analysis of quantitative reverse transcription polymerase chain reaction (qRT-PCR)

Total RNA was extracted from cells treated with RANKL and HX112 using TRIzol reagent (Invitrogen, USA), and then synthesized into cDNA using avian myeloblastosis virus reverse transcriptase (TaKaRa, Japan) and oligo primers (Qiagen, USA). The synthesized cDNA was amplified by qRT-PCR using Thermal Cycler Dice Real-Time System TP800 (TaKaRa, Japan) with specific primers shown in Table 1.

**Table 1:** Primer sequences.

Name		Sequence (5′ → 3′)
β-actin	Forward	AGG GAA ATC GTG CGT GAC AT
	Reverse	TCC AGG GAG GAA GAG GAT GC
NFATc1	Forward	GGA GAG TCC GAG AAT CGA GAT
	Reverse	TTG CAG CTA GGA AGT ACG TCT
c-Fos	Forward	ACT TCT TGT TTC CGG C
	Reverse	AGC TTC AGG GTA GGT G
MMP-9	Forward	GGA CCC GAA GCG GAC ATT G
	Reverse	GAA GGG ATA CCC GTC TCC GT
OC-STAMP	Forward	TGG GCC TCC ATA TGA CCT CGA GTA G
	Reverse	TCA AAG GCT TGT AAA TTG GAG GAG T
DC-STAMP	Forward	CCA AGG AGT CGT CCA TGA TT
	Reverse	GGC TGC TTT GAT CGT TTC TC

### 2.7 Western blotting

Total protein of RAW264.7 cells stimulated with RANKL and HX112 for either 30 min or 24 h was obtained using the T-PER tissue extraction reagent (Thermo Fisher, USA). Whole lysates were separated on Bolt™ Bis-Tris Plus Gels (Invitrogen, USA) and transferred to polyvinylidene fluoride (Cytiva, Korea). Then, membrane was blocked with 1X TBS Blocking buffer (Thermo Fisher, USA) and then incubated with a specific antibodies for NFATc1 (1:1000, Abcam, UK), c-FOS, p-Src, Src, p-PI3K, PI3K, p-AKT, AKT, p-JNK, JNK, p-p38, p38, p-ERK, ERK (1:1000, Cell Signaling, USA), and β-actin (1:5000, Sigma). Subsequently, HRP-conjugated anti-rabbit or anti-mouse IgG antibodies (1:5000, Cell Signaling) were additionally treated, and the target proteins were identified by enhanced chemiluminescence solution (Thermo Fisher) and ChemiDoc MP imaging system (Bio-Rad, USA).

### 2.8 Animal experiments

5-weeks-old female Institute of Cancer Research (ICR) mice were acquired from Orientbio Inc. (Korea). Mice were maintained under a 12 h light/dark cycle, and animal studies were conducted in accordance with the guidelines established by the Institutional Animal Care and Use Committee of Helixmith Co., Ltd. (approval number: VIC-20-08-001).

After acclimation for one week, ICR mice were ovariectomized. Ovariectomized mouse model is a well-established preclinical animal model mirroring human skeletal characteristics such as osteoporosis and osteopenia caused by estrogen deficiency [45,46]. Briefly, mice were anesthetized with isoflurane (Hana Pharm, Korea), and the bilateral ovaries were removed via the ventral approach, while the sham group underwent laparotomy. After 7 d of recuperation following surgery, animals were divided into six groups: Sham + vehicle (2% hydroxypropyl methylcellulose), OVX + vehicle, OVX + β-estradiol (Sigma, USA), OVX + HX112 150 mg/kg, OVX + HX112 300 mg/kg, and OVX + HX112 600 mg/kg (n=10). Each treatment was orally administered for 7 weeks on a daily basis. 8 weeks after ovariectomized, femur BMD was analyzed using micro-computed tomography (CT) scanning. After 3D bone structure evaluation, mice were euthanized, and the uterus weight was determined.

### 2.9 Bone structure evaluation using micro-CT

QUANTUM GXll micro-CT (Perkin Elmer, USA) was used to scan the mouse bone. The mice were anesthetized with isoflurane, placed in the sample bed of the scanner, and consecutive CT images were captured. The scanned 3D images were analyzed to calculate femur bone morphometric indices, such as BMD (mg/cm^3^, femur bone, cortex, trabecular, and intra-trabecular BMD), BV/TV (%, bone volume fraction), and BS/TV (mm^−1^, bone surface density) using the QUANTUM GXll micro-CT analyzer.

### 2.10 Statistical analysis

All data are mean ± standard error (SEM) accomplish from three independently experiments. Statistical analysis of this study was conducted one-way analysis of variance (ANOVA) with Tukey's or Dunnett's post hoc test in GraphPad Prism's (version 8.0; GraphPad Software, USA). A p-value less than 0.05 was considered statistically significant.

## 3 Results

### 3.1 Contents of marker compounds in HX112 assessed by HPLC analysis

**Fig. 1:**
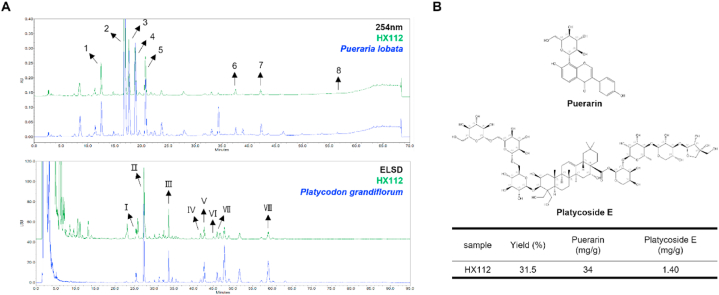
Chemical profile and content of marker compounds of HX112 as determined by HPLC analysis.

Each marker compound for *P*. *lobata* and *P*. *grandiflorum* was identified based on the literature and our previous studies as puerarin and platycoside E, respectively [47,48]. These marker compounds were detected in HX112 under our HPLC conditions (Fig. 1A, Supplementary Tables 1 and 2). As shown in Fig. 1B, the content of the extract used in this study was 34 mg/g for puerarin and 1.40 mg/g for platycoside E.

### 3.2 HX112 suppresses osteoclasts differentiation

**Fig. 2:**
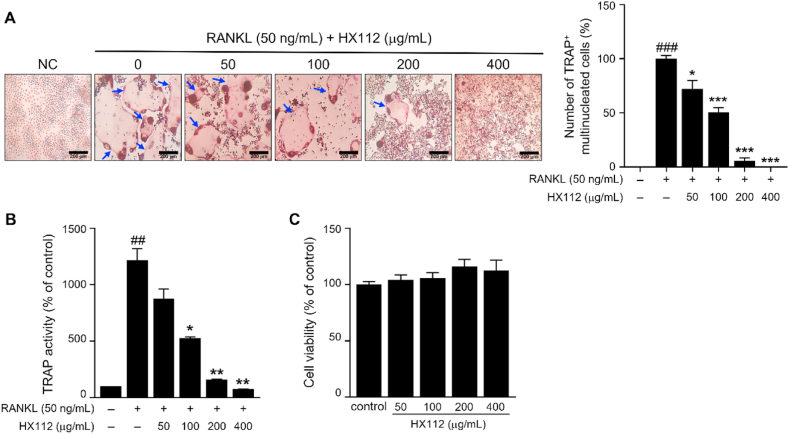
HX112 suppresses the RANKL-mediated differentiation of osteoclasts in RAW264.7 cells.

When exposed to RANKL, RAW264.7, a murine macrophage cell line, has the ability to differentiation into multinucleated cells like osteoclasts [49]. To investigate whether HX112 affects osteoclast differentiation, RAW264.7 cells were treated with RANKL and HX112 to induce osteoclast differentiation. As shown in Fig. 2A, RANKL treatment of RAW264.7 cells induced osteoclasts differentiation and increased the number of TRAP^+^ cells compared to the control group. On the other hand, HX112 treatment significantly decreased RANKL-induced osteoclasts differentiation in a dose-dependent manner (100 ± 6% vs 72.3 ± 14.9%, 50.4 ± 8.7%, 5.8 ± 4.9%, and 0%). In particular, the HX112 400 mg/mL treatment group decreased by almost 100% compared to the vehicle treatment group. Consistent with this, TRAP activity, which was increased by approximately 90% by RANKL, was markedly reduced by HX112 treatment (1217.2 ± 144.4% vs 875.8 ± 123%, 528 ± 14%, 159 ± 3.2%, and 74 ± 1.2%) (Fig. 2B). To determine whether HX112 has a cytotoxic effect, cell viability was evaluated using WST-1 assay kit. Cell viability was not impacted by any of the HX112 (Fig. 2C). These results indicated that HX112 suppresses the differentiation of osteoclast.

### 3.3 HX112 inhibits bone resorption

**Fig. 3:**
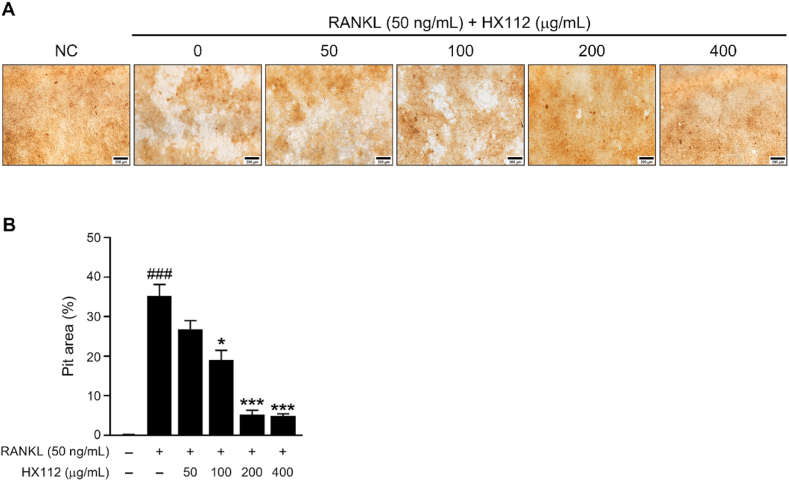
HX112 inhibits RANKL-mediated bone resorption in RAW264.7 cells.

Osteoclasts are involved in bone remodeling because they decompose and resorb bone [10]. Therefore, we hypothesized that HX112, which inhibits osteoclast differentiation, may also suppress bone resorption. To explore whether HX112 decreases bone resorption, cells were incubated on calcium phosphate-coated plates, and stimulated with HX112 and RANKL. Osteoclasts treated with RANKL actively absorbed and decomposed the calcium coated on the plate, forming pits (white region). On the other hand, osteoclasts cultured with HX112 had reduced calcium absorption and suppressed ability to form pits (Fig. 3A). As a result of analyzing the pit area, the pit area of osteoclasts incubated with HX112 was decreased by 90% compared to the pit area of osteoclasts treated with vehicle (35.2 ± 5.8% vs 26.8 ± 4.3%, 19 ± 4.9%, 5.2 ± 2.2%, and 4.9 ± 1.1%) (Fig. 3B). These data indicate that HX112 inhibits bone resorptive activity.

### 3.4 HX112 suppresses osteoclastogenic gene expression

**Fig. 4:**
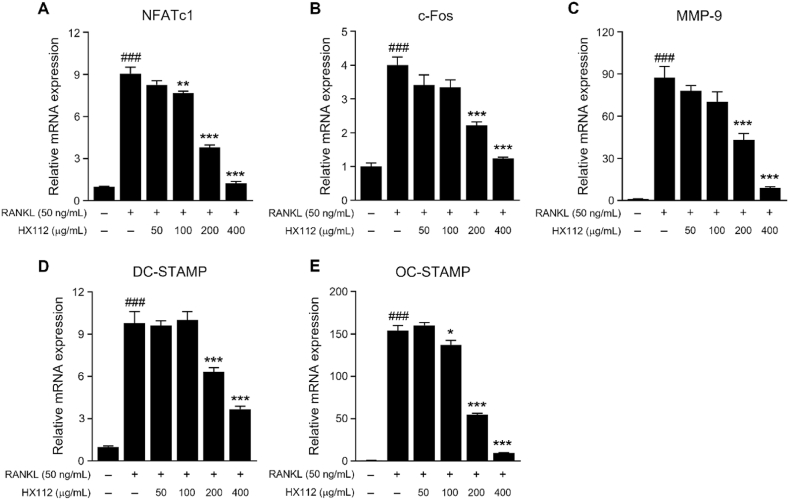
HX112 decreases expression of osteoclastogenic genes in RANKL-induced RAW264.7 cells.

NFATc1 is a key transcription factor involved in the generation of osteoclasts and bone resorption activity [13]. In addition, c-Fos is recognized as a key player in the osteoclast differentiation as an essential component of activator protein 1 (AP-1) that is activated by RANKL [50,51]. Therefore, we tested whether HX112 controlled osteoclastogenic genes expression. NFATc1 mRNA level, which was increased approximately 9-fold by RANKL, was notably decreased in a dose-dependent manner in HX112-treated osteoclasts (∼1.3 fold) (Fig. 4A). Additionally, the mRNA level of c-Fos increased approximately 4-fold by RANKL and then decreased to 1.2-fold by HX112 (Fig. 4B). Matrix metalloproteinase 9 (MMP9) mRNA expression level, which promotes bone resorption, also increased 85-fold with RANKL treatment, but was significantly decreased with HX112 treatment (∼9-fold) (Fig. 4C). Furthermore, the mRNA expression of dendritic cell-specific transmembrane protein (DC-STAMP), which promotes cell-cell fusion, and osteoclast stimulatory transmembrane protein (OC-STAMP), which develops the differentiation of osteoclast, was noticeably decreased in HX112-treated cells compared to vehicle-treated cells (9.8-fold vs ∼3.7-fold, 153.8-fold vs ∼9.5-fold, respectively) (Fig. 4D and E). These data showed that HX112 suppresses osteoclastogenic genes expression.

### 3.5 HX112 inhibits Src-PI3K-AKT and JNK/p38 signaling pathways

**Fig. 5:**
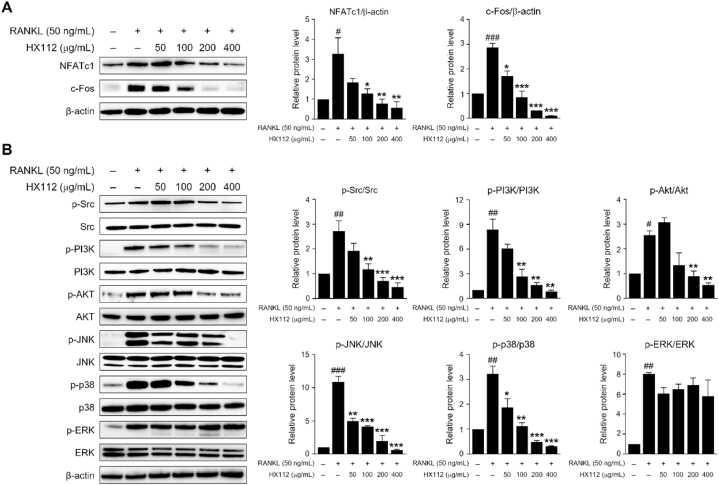
HX112 inhibits the expression of NFATc1 and c-Fos by regulating Src-PI3K-Akt and JNK/p38 signaling pathways.

Next, we examined whether HX112 controls NFATc1 and c-Fos protein production. NFATc1 protein levels, which increased by about 3.3-fold by RANKL, decreased by 83% by HX112 treatment (∼0.6-fold). The protein level of c-Fos also increased by almost 2.9-fold by RANKL, but diminished by approximately 97% when incubated with HX112 (∼0.1-fold) (Fig. 5A).

To explore whether HX112 can regulate signaling pathways associated with RANKL, cells incubated with HX112 and RANKL for 30 min and then analyzed then by Western blot. The phosphorylation of PI3K and Akt increased by RANKL was remarkably reduced by HX112 treatment (8.4-fold vs ∼0.86-fold and 2.6-fold vs ∼0.5-fold, respectively) (Fig. 5B).

Activation of the PI3K-Akt signaling pathway in osteoclasts is widely recognized to be reliant on the activity of Src kinase [52]. Therefore, we investigated whether HX112 regulates Src kinase phosphorylation. As a result, we observed that the increased phosphorylation of Src by RANKL was attenuated by about 85% in the presence of HX112 (2.7-fold vs ∼0.5-fold) (Fig. 5B). These data showed that HX112 suppresses the PI3K-Akt axis by inhibiting Src activity. Furthermore, RANKL stimulation markedly enhanced the phosphorylation of JNK and p38, but these effects were clearly decreased when co-treated with HX112 (10.9-fold vs ∼0.6-fold and 3.2-fold vs ∼0.3-fold, respectively). On the other hand, HX112 had no effect on the increased phosphorylation of ERK by RANKL treatment (8-fold vs ∼5.8-fold) (Fig. 5B). Collectively, our results suggest that HX112 regulates the activity of Src-PI3K-Akt and JNK/p38 signaling pathways.

### 3.6 HX112 alleviates ovariectomy-induced bone loss

**Fig. 6:**
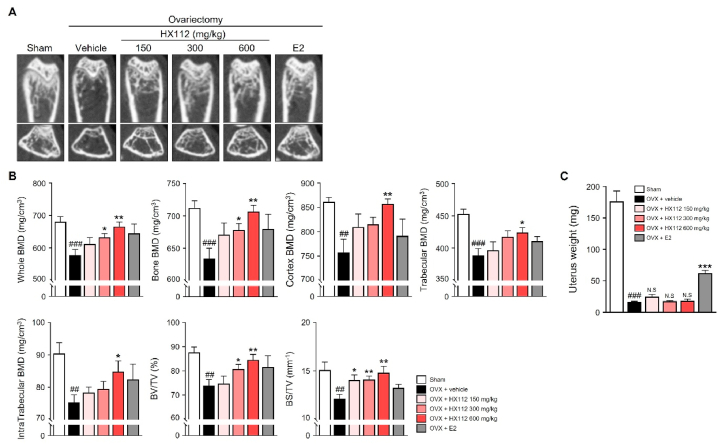
HX112 prevents ovariectomy-induced bone loss in mice.

Based on the above data, we expected that HX112 would have therapeutic effects in protecting against osteoporosis. To investigate this possibility, we employed an ovariectomized mouse model. HX112 was orally administered daily after ovarian resection, and femurs were analyzed eight weeks later using micro-CT. As shown Fig. 6A, the bone microstructure of the ovariectomized mice was markedly diminished compared to that of the sham control mice, whereas it was significantly recovered in mice fed HX112. Consistent with Fig. 6A, femur BMD, including whole BMD, bone BMD, cortical BMD, trabecular BMD, and intratrabecular BMD was notably reduced in vehicle-treated ovariectomized mice. While, all femur BMD were significantly improved in mice administered HX112 (577.4 ± 56.9 mg/cm^3^ vs 665.8 ± 39.8 mg/cm^3^, 634 ± 50.4 mg/cm^3^ vs 706.8 ± 26.7 mg/cm^3^, 757.7 ± 85 mg/cm^3^ vs 857.2 ± 29.5 mg/cm^3^, 388.5 ± 34.6 mg/cm^3^ vs 424.2 ± 21.2 mg/cm^3^ and 75.3 ± 7.4 mg/cm^3^ vs 84.8 ± 9.4 mg/cm^3^, respectively). Futhermore, BV/TV and BS/TV, which are morphological indicators of trabecular bone, were lowered to 73.9 ± 7.8% and 12.2 ± 1.4 mm^−1^, respectively, in the vehicle-administered group, but improved to 84.5 ± 6.5% and 14.8 ± 1.9 mm^−1^. by HX112 administration (Fig. 6B). To determine whether HX112 may have an estrogen-responsive cancer risk, uterus weight was measured. Estrogen-administered mice showed a marked increase in uterine weight, whereas there were no changes in HX112-administered mice compared to vehicle-administered mice (Fig. 6C). Taken together, these data demonstrate that HX112 ameliorates ovariectomy-induced bone loss without estrogen-responsive cancer risk.

## 4 Discussion

In this study, we proved that HX112 inhibits RANKL-mediated osteoclastogenesis. HX112 decreased TRAP^+^ multinucleated cells number, reduced the activity of TRAP, and inhibited pit formation. In addition, HX112 reduced the osteoclastogenesis-related gene expression, and suppressed the Src-PI3K-Akt and JNK/p38 signaling pathways activated by RANKL. Furthermore, it has been shown that HX112 can effectively inhibit bone loss resulting from ovariectomy by restoring BMD and destroying the microstructure of femurs.

TNF receptor associated factor 6 (TRAF6) predominantly regulates RANKL-mediated MAPK activation. TRAF6 is an adapter protein belonging to the TRAF family, and its role in RANKL-induced osteoclast differentiation has been previously reported. When RANKL binds to RANK, TRAF6 is recruited and complexed with MEKK3 to activate p38 and JNK (but not ERK) [53]. TRAF6 also contributes to Transforming growth factor beta-activated kinase 1 (TAK1) activation, which in turn upregulates AP-1 by regulating the MKK7-JNK and MKK6-p38 pathways [53,54]. Moreover, it has previously been reported that MEKK3 is likely activated by TAK1 [55]. In our results, HX112 inhibited p38 and JNK activation but it had no impact on the activity of ERK, which is similar to the function of TRAF6 reported in previous studies. Because puerarin, a major component of HX112, is known to suppress osteoclastogenesis by inhibiting the TRAF6-MAPK signaling pathway [56], HX112 may also exert its therapeutic effects by regulating the activity of TRAF6 or its downstream kinases MEKK3 and/or TAK1.

As postmenopausal syndrome is directly caused by a decrease in estrogen production, significant improvement in symptoms can be expected when hormone replacement therapy with synthetic 17β-estradiol is administered [57]. However, it has been reported that the risk of estrogen-dependent cancer, including ovarian and womb cancer, increases in patients who have received such hormonal therapy because synthetic estrogen binds to estrogen receptor (ER) α with higher affinity than ERβ [[58], [59], [60]]. To solve the problems associated with these current treatments, phytoestrogen-based therapy has recently been implemented for menopausal patients. Unlike synthetic estrogens, phytoestrogens have a high binding affinity for ERβ, which inhibits mammalian cell growth and thus has a high safety profile [61]. In addition, phytoestrogens contribute to symptom relief in postmenopausal syndrome through independent mechanisms, such as anti-oxidative stress activity and ERβ activity [62]. Phytoestrogens are classified into two classes: isoflavones and lignan [63]. Isoflavones are known to exhibit a high estrogenic effect based on a chemical structure similar to that of 17β-estradiol [63]. Since puerarin [64], daidzin [65], daidzein [66], ononin, and mirificin [67], which constitute most of the chemical compounds contained in HX112, are all isoflavone compounds, HX112 is expected to show higher safety by activating ERβ than ERα. This possibility is supported by the fact that uterine weight increased significantly in the estrogen-administered group [[68], [69], [70]], whereas no change was observed in the HX112-administered group, as shown in Fig. 6C. However, this study has limitations in that it has not yet investigated the combined effects of the various components contained in HX112 on the activities of ERα and ERβ. To directly evaluate the binding affinity of a mixture of compounds constituting HX112 to ERα and ERβ, a docking simulation study is currently in progress.

Osteoporosis can be caused not only by increased osteoclast differentiation but also by decreased osteoblast differentiation. Osteoblasts produce extracellular matrix proteins and bone morphogenetic factors that trigger bone formation, promote mineralization of the extracellular matrix, and consequently increase bone formation [71]. Various estrogen-related signaling pathways are involved in osteoblast differentiation. Estrogen directly inhibits RANKL expression and increases the expression of osteoprotegerin, a decoy receptor for RANKL [72]. Estrogen promotes the Bone morphogenetic protein 4 (BMP4)-Smad 1/5/8 signaling pathway, a key axis of osteoblast differentiation, by suppressing Smad 6/7 activity [73]. In addition, estrogen acts synergistically with Wnt3A to enhance osteogenic differentiation [74]. In this context, because HX112 contains various chemical compounds exhibiting estrogenic activity, it is possible that HX112 promotes bone formation by regulating osteoblast differentiation. For example, puerarin, the marker compound present in the highest amount in HX112, promotes osteoblast differentiation through activation of the NO/cGMP pathway [75]. In addition, platycodin D, a major component of *P. grandiflorum*, increases key transcription factors expression involved in osteoblastogenesis, such as RUNX2 and osterix, through the GSK3β/β-catenin signaling pathway [76]. Platycodin E, the main component of HX112, is hydrolyzed and converted to platycodin D, which is expected to exhibit anti-osteoporotic activity [77]. The possible mechanisms by which HX112 regulate osteoblast differentiation are currently being explored.

## 5 Conclusion

In conclusion, HX112 suppressed osteoclasts differentiation and function through Src-PI3K-Akt and JNK/p38 signaling pathway and consequently alleviate osteoporosis. Our study results demonstrate that HX112 exhibits considerable promise as a therapeutic option for treatment osteoporosis in menopausal women, due to various mechanisms of action and high safety identified in this study. To verify this possibility, clinical trial for menopausal women is currently being conducted in Korea.

## Data availability statement

Data included in article/supplementary material/referenced in article.

## CRediT authorship contribution statement

**Jisun Song:** Writing – review & editing, Writing – original draft, Project administration, Investigation. **Suhyun Han:** Investigation. **Sooyeon Choi:** Investigation. **Jungkyu Lee:** Investigation. **Yoonseon Jeong:** Investigation. **Hyun Myung Lee:** Investigation. **JongDai Son:** Investigation. **Dam Yeon Jeong:** Investigation. **Seung-Shin Yu:** Project administration, Data curation. **Wonwoo Lee:** Writing – review & editing, Writing – original draft, Project administration, Investigation, Data curation.

## Declaration of competing interest

The authors declare the following financial interests/personal relationships which may be considered as potential competing interests:Jisun Song reports a relationship with Helixmith Co Ltd that includes: employment. Suhyun Han reports a relationship with Helixmith Co Ltd that includes: employment. Sooyeon Choi reports a relationship with Helixmith Co Ltd that includes: employment. Jungkyu Lee reports a relationship with Helixmith Co Ltd that includes: employment. Yoonseon Jeong reports a relationship with Helixmith Co Ltd that includes: employment. Hyun Myung Lee reports a relationship with Helixmith Co Ltd that includes: employment. JongDai Son reports a relationship with Helixmith Co Ltd that includes: employment. Dam Yeon Jeong reports a relationship with Helixmith Co Ltd that includes: employment. Seung-Shin Yu reports a relationship with Helixmith Co Ltd that includes: employment. Wonwoo Lee reports a relationship with Helixmith Co Ltd that includes: employment.
