# Supplementary material for: A mixture of Pueraria lobata and Platycodon grandiflorum extracts ameliorates RANKL-induced osteoclast differentiation and ovariectomy-induced bone loss by regulating Src- PI3K-AKT and JNK/p38 signaling pathways
Source: Heliyon. 2024 Jan 17;10(2):e24842. doi: 10.1016/j.heliyon.2024.e24842 (PMC10835310; doi:10.1016/j.heliyon.2024.e24842)

Supplementary Table 1. Analysis method for chemical profile of *Pueraria lobata*

Instrument	Waters Alliance 2695 HPLC
Detector	Photodiode Array Detector (PDA)
Wavelength	254 nm
Column	YoungJin Biochrom INNO-HC C18 (150 mm x 4.6 mm, 5 µm)
Mobile phase	A: 0.1% Formic acid in Water<br>B: 0.1% Formic acid in Methanol
Flow rate	1.0 mL/min
Injection volume	10 µL
Temperature	25℃ (Oven), 15℃ (Autosampler)

Mobile phase gradient:

Time (min)	A (%)	B (%)
0	77	23
5	77	23
13	67	33
26	61	39
35	50	50
45	47	53
55	30	70
60	0	100
65	0	100
65.1	77	23
70	77	23

Supplementary Table 2. Analysis method for chemical profile of *Platycodon grandiflorum*

Instrument	Waters Alliance 2695 HPLC
Detector	Evaporative Light Scattering Detector (ELSD)
ELSD parameter	Gain: 100, Gas pressure: 45 psi, Nebulizer: cooling, Drift tube: 80℃
Column	YoungJin Biochrom INNO C18 (150 mm x 4.6 mm, 3.5 µm)
Mobile phase	A: 30mM Ammonium acetate (0.07% FA): MeOH: ACN = 80:5:15<br>B: 30% Acetonitrile

Mobile phase gradient:

Time (min)	A (%)	B (%)
0	85	15
5	85	15
15	75	25
20	65	40
30	30	70
50	30	70
60	20	80
70	0	100
85	0	100
85.1	85	15
95	85	15

Flow rate	0.7 mL/min
Injection volume	20 μL
Temperature	40℃ (Oven), 15℃ (Autosampler)

Supplementary Figure 1. Original blot of changes in expression of NFATc1 and c-Fos by HX112

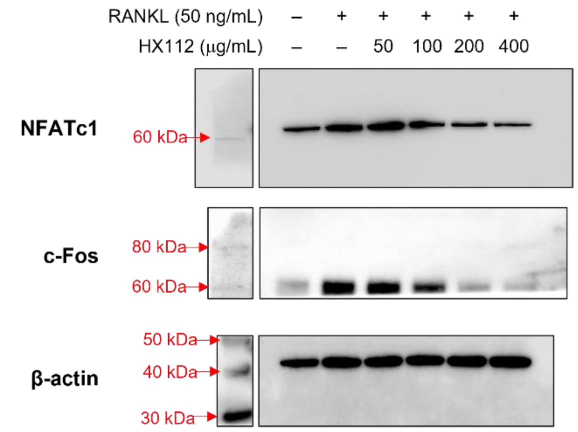

Supplementary Figure 2. Original blot of changes in signaling pathway activation by HX112

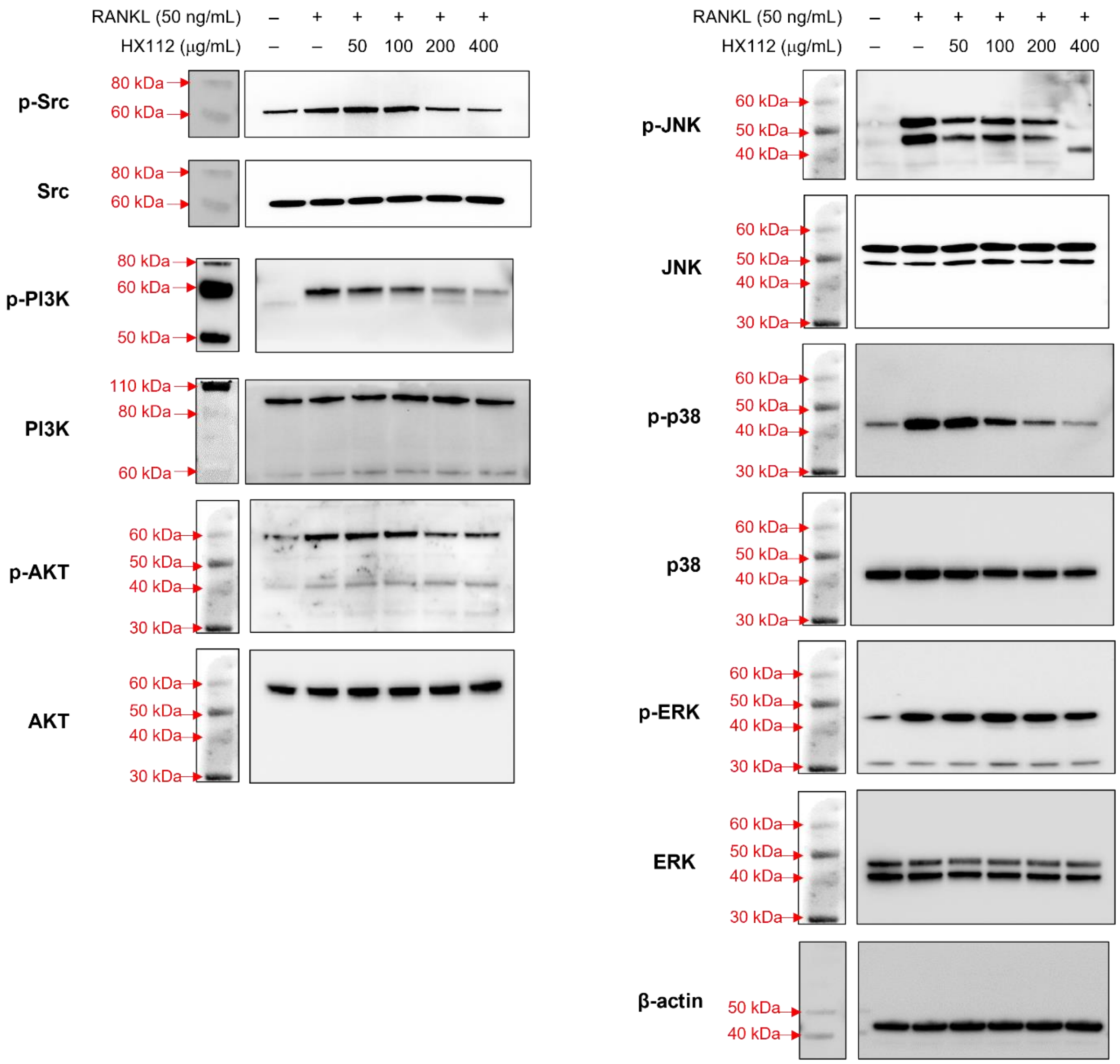

Supplement: Multimedia component 1 [file mmc1.pdf]
